# IGK with conserved IGKV/IGKJ repertoire is expressed in acute myeloid leukemia and promotes leukemic cell migration

**DOI:** 10.18632/oncotarget.5393

**Published:** 2015-09-29

**Authors:** Chong Wang, Miaoran Xia, Xiaoping Sun, Zhiqiao He, Fanlei Hu, Lei Chen, Carlos E. Bueso-Ramos, Xiaoyan Qiu, C. Cameron Yin

**Affiliations:** ^1^ Department of Immunology, School of Basic Medical Sciences, Peking University, Beijing, China; ^2^ Department of Hematopathology, UT MD Anderson Cancer Center, Houston, TX, USA; ^3^ Department of Laboratory Medicine, UT MD Anderson Cancer Center, Houston, TX, USA; ^4^ Department of Pathology, University of Texas-Houston Medical School, Houston, TX, USA

**Keywords:** IgK, acute myeloid leukemia, restricted IGKV/IGKJ rearrangement, somatic hypermutation, leukemic cell migration

## Abstract

We have previously reported that immunoglobulin heavy chain genes were expressed in myeloblasts and mature myeloid cells. In this study, we further demonstrated that rearranged Ig κ light chain was also frequently expressed in acute myeloid leukemia cell lines (6/6), primary myeloblasts from patients with acute myeloid leukemia (17/18), and mature monocytes (11/12) and neutrophils (3/12) from patients with non-hematopoietic neoplasms, but not or only rarely expressed in mature neutrophils (0/8) or monocytes (1/8) from healthy individuals. Interestingly, myeloblasts and mature monocytes/neutrophils shared several restricted IGKV and IGKJ gene usages but with different expression frequency. Surprisingly, almost all of the acute myeloid leukemia-derived IGKV showed somatic hypermutation; in contrast, mature myeloid cells-derived IGKV rarely had somatic hypermutation. More importantly, although IGK expression appeared not to affect cell proliferation, reduced IGK expression led to a decrease in cell migration in acute myeloid leukemia cell lines HL-60 and NB4, whereas increased IGK expression promoted their motility. In summary, IGK is expressed in myeloblasts and mature myeloid cells from patients with non-hematopoietic neoplasms, and is involved in cell migration. These results suggest that myeloid cells-derived IgK may have a role in leukemogenesis and may serve as a novel tumor marker for monitoring minimal residual disease and developing target therapy.

## INTRODUCTION

It has been assumed that immunoglobulin (Ig) can only be produced by B-cells or plasma cells, but not other types of cells. However, during the last decade, this concept has been challenged by a series of studies. Initially, it was found that Ig, including IgG, IgM and IgA, was expressed in many epithelial cancer cells, including breast, colon, lung, liver, cervical and oral cancers [1–6]. Subsequently, Ig was also found in normal non-hematopoietic cells, including epithelium, germ cells, neuron and myocardial cells [7–10]. Moreover, Ig produced by non-hematopoietic cells showed some unique characteristics. For example, unlike B-cell-derived Ig that shows great diversity, non-B-cell-derived Ig repertoire did not show diversity, but demonstrated unique usage and sequence [9, 11]. In addition, it had unique glycosylation profile [12] and a regulatory mechanism that was different from that in B-cells [13–15]. More importantly, growing evidence has revealed that non-B-cell-derived Ig is involved in cell survival and carcinogenesis [1, 4].

More recently, we have reported that Ig γ and μ genes can be expressed in myeloid cells [16, 17]. The IgG was frequently expressed in acute myeloid leukemia (AML) cells (myeloblasts), but not in monocytes and neutrophils, and was involved in the survival of myeloblasts [16]. The IgM was expressed in both myeloblasts and monocytes and neutrophils with a similar frequency, and its expression was involved in cell proliferation [17]. To date, it remains unclear if Ig light chain is expressed in myeloid cells, and if it is, what is its significance in leukemogenesis.

In this study, we have for the first time confirmed that Ig κ light chain (IGK) was frequently expressed in AML cell lines and primary myeloblasts, but not or rare in monocytes and neutrophils from healthy individuals. Surprisingly, IGK was also detected in monocytes and neutrophils from patients with non-hematopoietic neoplasms. Moreover, almost all AML-derived IGKV showed somatic hypermutation, whereas virtually no mutation was detected in mature myeloid cell-derived IGKV. These results suggest that IGK can be expressed by myeloid cells, and that IGKV somatic hypermutation may be involved in leukemogenesis. We further studied the function of IGK expression by knocking down or overexpressing IGK in AML cell lines, and found that IgK was mainly involved in the migration of AML cells.

## RESULTS

### IGK is frequently expressed in AML cell lines and primary myeloblasts

**Figure 1:**
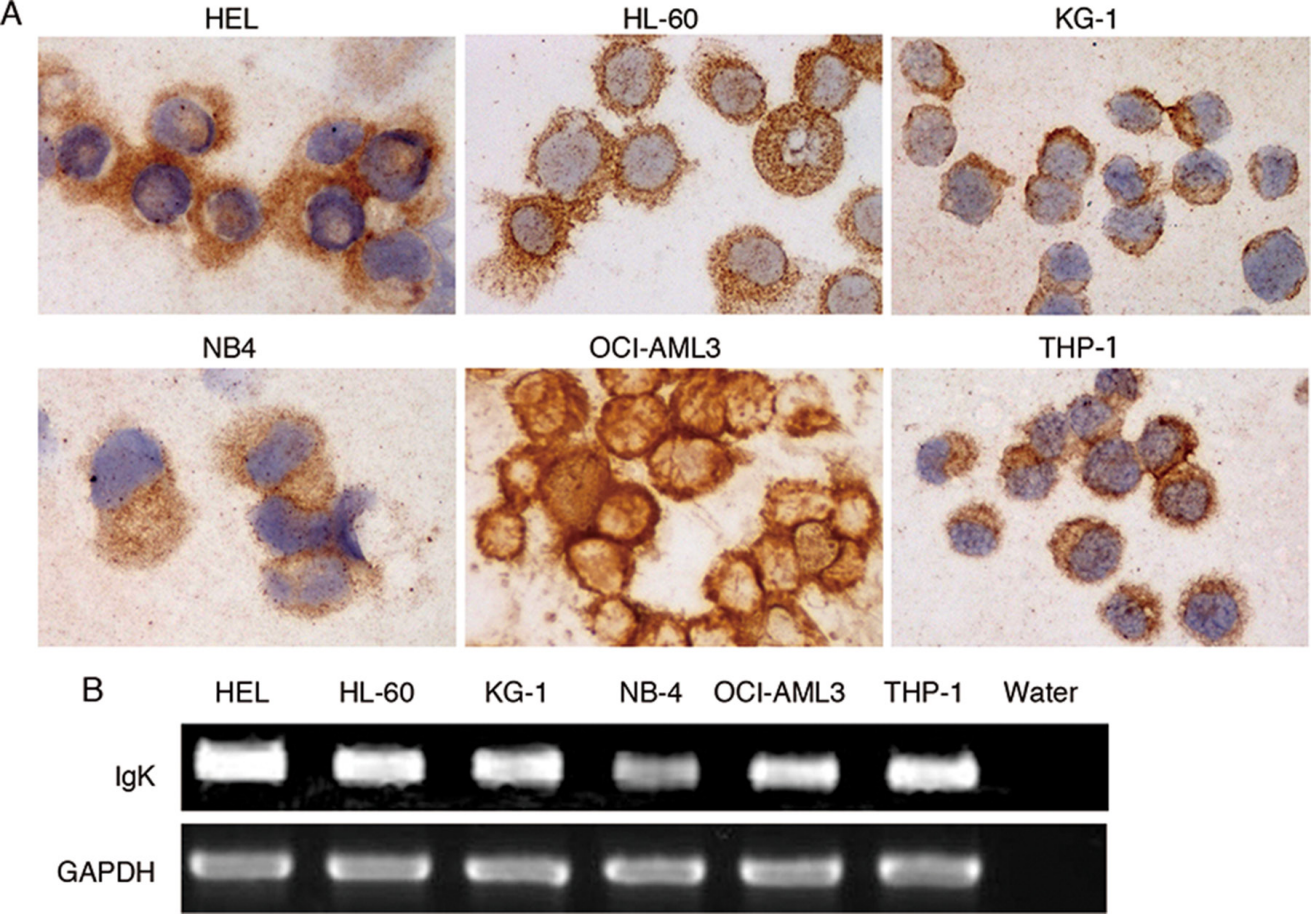
IGK expression in AML cell lines **A.** Immunocytochemical study showed IGK expression in all 6 AML cell lines. **B.** RT-PCR analysis detected IGK transcripts in all 6 AML cell lines.

To test if IGK is expressed in AML cells, we performed immunocytochemical studies using monoclonal anit-human IgK in 6 AML cell lines, HEL, HL-60, KG-1, NB4, OCI-AML3 and THP-1, and found strong cytoplasmic staining in all 6 AML cell lines (Figure 1A). This was in contrast to the staining pattern seen in B-lymphoma cell line, SP53, which mainly showed membrane staining (data not shown). Next, we performed RT-PCR followed by sequencing and confirmed that IGK was rearranged and transcribed in all 6 AML cell lines (Figure 1B).

**Figure 2:**
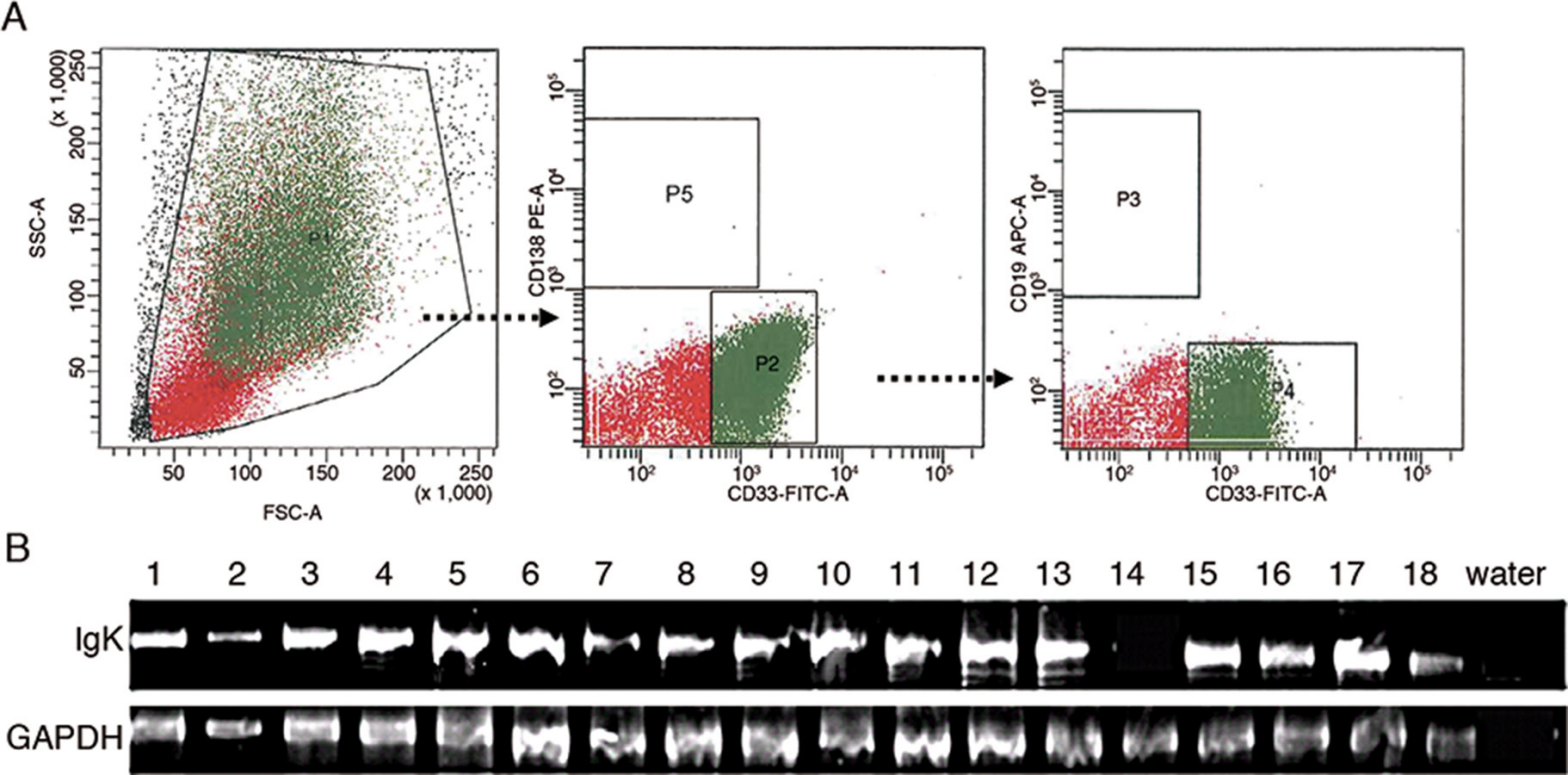
IGK expression in primary myeloblasts **A.** Flow cytometry sorting of myeloblasts. P1: mononuclear cells; P2: CD33^+^CD138^−^ cells from P1; P3: CD19^+^ cells from P2; P4: CD33^+^CD19^−^cells from P2; P5: CD138^+^ plasma cells. The cells in P4 were defined as CD33^+^CD19^−^CD138^−^ myeloblasts. The cells in P3 were used as a positive control. **B.** RT-PCR analysis detected IGK transcripts in myeloblasts from 17 of 18 AML patients.

Furthermore, to study if IGK was also expressed in primary myeloblasts, we performed RT-PCR on sorted CD33^+^CD19^−^CD138^−^ myeloblasts from peripheral blood of 18 AML patients (Figure 2A). Sorted CD19^+^ B-cells from the same individuals were used as a positive control. We detected rearranged transcripts of IGK in myeloblasts from 17 of 18 AML patients (Figure 2B).

### IGK is expressed in mature monocytes and neutrophils from patients with non-hematopoietic neoplasms, but not or rarely from health individuals

**Figure 3:**
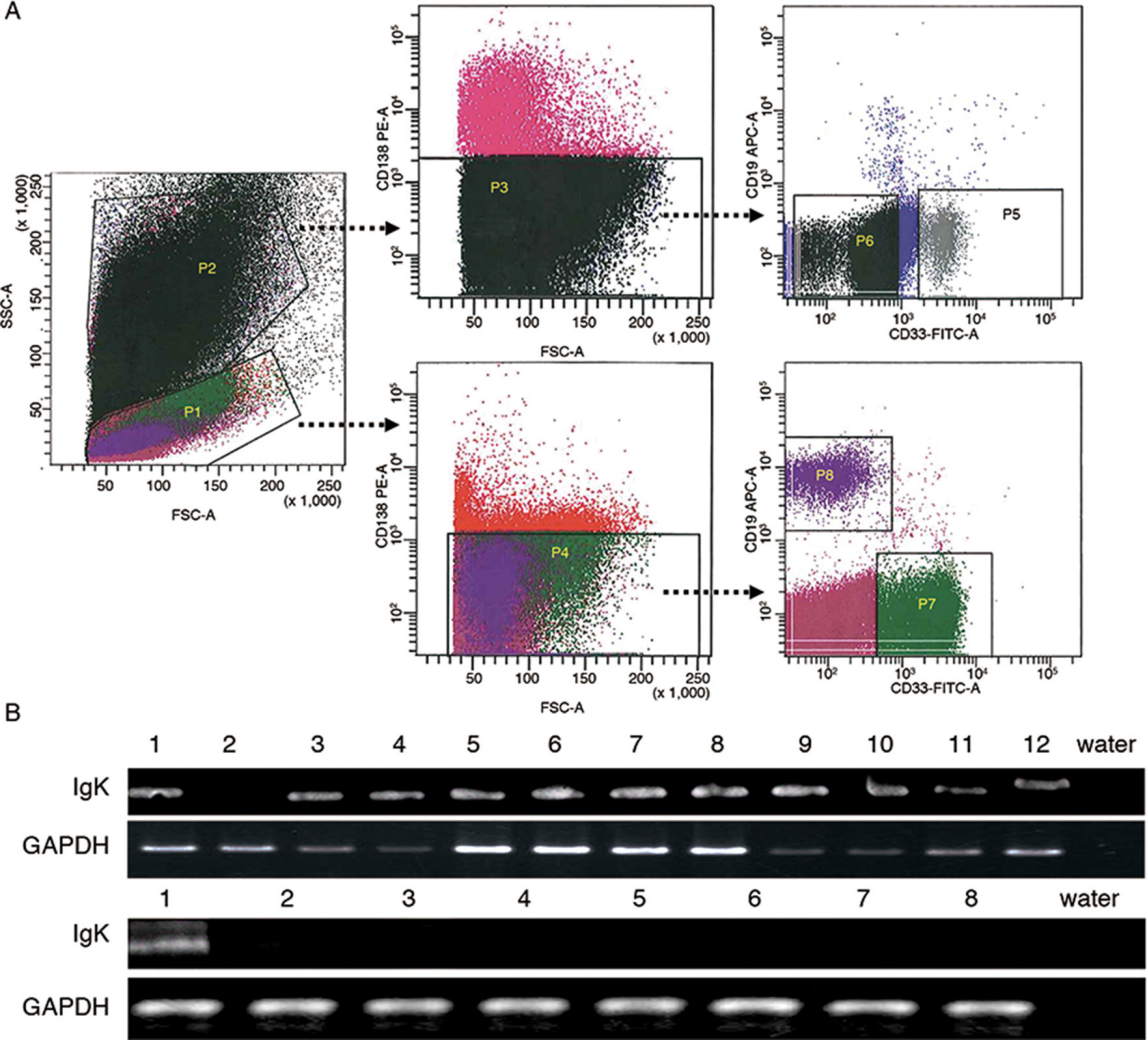
IGK expression in monocytes and neutrophils from patients with non-hematopoietic neoplasms and healthy individuals **A.** Flow cytometry sorting of monocytes and neutrophils. P1: mononuclear cells; P2: granulocytes; P3: CD138^−^ granulocytes from P2; P4: CD138^−^ mononuclear cells from P1; P5: CD33^+^ CD19^−^ neutrophils from P3; P6: CD33^−/dim+^CD19^−^ granulocytes from P3; P7: CD33^+^CD19^−^ monocytes from P4; P8: CD19^+^ B-cells from P4. Cells in P5, P7 and P8 were collected. **B.** RT-PCR analysis detected IGK expression in the monocytes from 11 of 12 patients with non-hematopoietic neoplasms (upper panel), but only in the monocytes from 1 of 8 healthy individuals (lower panel).

To address if IGK is expressed in mature myeloid cells, we sorted CD33^+^CD19^−^CD138^−^ neutrophils (Figure 3A, upper panel) and monocytes (Figure 3A, lower panel) from 12 patients with non-hematopoietic neoplasms (including 4 cases of colorectal adenocarcinoma, 2 cases of stomach adenocarcinoma, 2 cases of squamous cell carcinoma, and 1 case each of pancreatic adenocarcinoma, high-grade sarcoma, glioblastoma and thymoma), as well as 8 healthy individuals. Unlike IGG that was only expressed in AML cells but not in mature myeloid cells [16], IGKV/IGKJ rearrangements were also detected in monocytes from 11/12 patients (Figure 3B, upper panel) and in neutrophils from 3/12 patients with non-hematopoietic neoplasms. However, IGKV/IGKJ rearrangements were not or only rarely detected in neutrophils (0/8) or monocytes (1/8, Figure 3B, lower panel) from healthy individuals.

### AML-derived IGK shows a monoclonal or oligoclonal IGKV/IGKJ rearrangement pattern

Furthermore, we analyzed IGKV/IGKJ rearrange-ments of AML-derived IGK, and detected IGK transcripts with monoclonal or oligoclonal IGKV/IGKJ rearrangements in all of the 5 AML cell lines assessed (HEL, NB4, OCI-AML3, THP-1 and KG-1). However, different IGKV/IGKJ usages were detected in different cell lines, with the exception that IGKV1-NL1*01/IGKJ5*01 was expressed by 3 of 5 AML cell lines, including HEL, NB4 and KG-1 (Table 1).

**Table 1:** The IGKV/IGKJ rearrangement patterns in AML cell lines

AML cell lines	IGKV/IGKJ usage
HEL	IGKV3-20*01/IGKJ3*01 (1/2)
	IGKV1-NL1*01/IGKJ5*01 (1/2)
NB4	IGKV1-NL1*01/IGKJ5*01 (5/5)
OCI-AML3	IGKV3D-7*01/IGKJ4*01 (1/1)
THP-1	IGKV3-NL5*01/IGKJ1*01 (1/1)
KG-1	IGKV1-NL1*01/IGKJ5*01 (3/5)
	IGKV3D-20*01/IGKJ2*01 (2/5)

We subsequently analyzed AML-derived IGKV/IGKJ rearrangements from the 17 AML cases. Unlike CD19^+^ B-cell-derived IGKV/IGKJ rearrangements that showed diversity, we found that myeloblast-derived IGKV/IGKJ rearrangements showed monoclonal or oligoclonal patterns in each individual. Only 15 IGKV/IGKJ rearrangement patterns were displayed in a total of 104 clones assessed. Notably, 3 sets of IGKV/IGKJ rearrangements occurred most frequently in the myeloblasts: IGKV1-5*03/IGKJ3*01 (53/77 clones from 12/17 cases), IGKV1-5*03/IGKJ1*01 (10/28 clones from 4/17 cases) and IGKV1-NL1*01/IGKJ5*01 (11/16 clones from 3/17 cases) (Table 2). Surprisingly, the IGKV1-5*03/IGKJ3*01 sequence from different cases exhibited 98–100% homology, while the IGKV1-NL1*01/IGJ5*01 sequence from either different AML patients or AML cells lines exhibited 100% homology (Supplementary Figure 1).

**Table 2:** The IGKV/IGKJ rearrangement patterns in primary myeloblasts from AML patients

Case#	IGKV/IGKJ usage	Case#	IGKV/IGKJ usage
1	IGKV3-15*01/IGKJ1*01 (5/7)	10	IGKV1-5*03/IGKJ3*01 (6/9)
	IGKV1D-39*01/IGKJ1*01 (2/7)		IGKV1-5*03/IGKJ1*01 (3/9)
2	IGKV1-5*03/IGKJ3*01 (4/8)	11	IGKV1-NL1*01/IGKJ5*01 (2/5)
	IGKV1-9*01/IGKJ3*01 (3/8)		IGKV1-5*03/IGKJ3*01 (2/5)
	IGKV3-11*01/IGKJ1*01 (1/8)		IGKV3-11*01/IGKJ2*01 (1/5)
3	IGKV1-5*03/IGKJ3*01 (4/8)	12	IGKV1-5*03/IGKJ3*01 (5/8)
	IGKV1-5*01/IGKJ3*01 (1/8)		IGKV2D-28*01/IGKJ3*01 (1/8)
	IGKV2D-28*01/IGKJ3*01 (1/8)		IGKV1-9*01/IGKJ3*01 (1/8)
	IGKV1-27*01/IGKJ1*01 (1/8)		IGKV2-24*01/IGKJ2*01 (1/8)
	IGKV2-24*01/IGKJ2*01 (1/8)		
4	IGKV1D-39*01/IGKJ1*01 (3/6)	13	IGKV1-5*03/IGKJ1*01 (2/7)
	IGKV1-5*03/IGKJ1*01 (3/6)		IGKV1D-39*01/IGKJ1*01 (2/7)
			IGKV1D-39*01/IGKJ1*04 (2/7)
			IGKV3-20*01/IGKJ4*01 (1/7)
5	IGKV1-5*03/IGKJ3*01 (8/8)	14	IGKV3-20*01/IGKJ4*01 (2/2)
6	IGKV1-5*03/IGKJ3*01 (5/5)	15	IGKV1-NL1*01/IGKJ5*01 (5/5)
7	IGKV1-5*03/IGKJ3*01 (6/6)	16	IGKV1-5*03/IGKJ3*01 (4/6)
			IGKV1-5*03/IGKJ1*01 (2/6)
8	IGKV1-5*03/IGKJ3*01 (3/4)	17	IGKV1-NL1*01/IGKJ5*01 (4/6)
	IGKV1-5*03/IGKJ5*01 (1/4)		IGKV1-5*03/IGKJ3*01 (2/6)
9	IGKV1-5*03 /IGKJ3*01 (4/4)		

### Mature myeloid cell-derived IGK displays a monoclonal or oligoclonal rearrangement pattern

Similarly, we analyzed 84 clones of IGKV/IGKJ rearrangements in mature monocytes (9 cases) and neutrophils (3 cases) from patients with non-hematopoietic neoplasms. The IGKV/IGKJ rearrangements also revealed biased IGKV/IGKJ patterns in each individual. Twelve IGKV/IGKJ rearrangements were found in 84 clones, 5 sets of IGKV/IGKJ rearrangements, IGKV3-20*01/IGKJ1*01 (18/23 clones from 2/9 cases of monocytes and 1/3 cases of neutrophils), IGKV3-20*01/IGKJ3*01 (7/16 clones from 1/9 cases of monocytes and 1/3 cases of neutrophils), IGKV1-27*01/IGKJ1*01 (10/12 clones from 1/9 cases of monocytes and 1/3 cases of neutrophils), IGKV1-27*01/IGKJ4*01 (14/14 clones from 1/9 cases of monocytes and 1/3 cases of neutrophils) and IGKV1-5*03/IGKJ3*01 (11/16 clones from 2/9 cases of monocytes), were frequently detected in mature myeloid cells (Table 3).

**Table 3:** The IGKV/IGKJ rearrangement patterns in monocytes and neutrophils

Case#	IGKV/IGKJ usage in monocytes	IGKV/IGKJ usage in neutrophils
1^a^	IGKV1-27*01/IGKJ4*01 (7/7)	IGKV1-27*01/IGKJ4*01 (7/7)
2	IGKV3-20*01/IGKJ1*01 (2/5)	IGKV1-27*01/IGKJ1*01 (6/8)
	IGKV3-15*01/IGKJ4*01 (2/5)	IGKV3-20*01/IGKJ3*01 (2/8))
	IGKV1D-16*02/IGKJ2*01 (1/5)	
3	ND	IGKV3-20*01/IGKJ1*01 (8/8)
4	IGKV1-27*01/IGKJ1*01 (4/4)	ND
5	IGKV1-5*01/IGKJ1*01 (6/6)	ND
6	IGKV1-5*03/IGKJ3*01 (8/8)	ND
7	IGKV1-9*01/IGKJ4*01 (6/6)	ND
8	IGKV3-20*01/IGKJ1*01 (8/10)	ND
	IGKV1-8*01/IGKJ4*01 (2/10)	
9	IGKV3-20*01/IGKJ3*01 (5/8)	ND
	IGKV1-5*03/IGKJ3*01 (3/8)	
10	IGKV4-1*01/IGKJ2*01 (5/7)	ND
	IGKV4-1*01/IGKJ1*01 (2/7)	

a 89% homology between monocytes and neutrophils

### CD33^+^ myeloblasts and mature myeloid cells demonstrate similar IGKV usage but with different frequencies

We compared IGKV/IGKJ rearrangement patterns in CD33^+^ myeloblasts and mature myeloid cells and found that both myeloblasts and mature myeloid cells shared 6 IGKV usages – IGKV1-5*01, IGKV1-5*03, IGKV1-9*01, IGKV1-27*01, IGKV3-15*01 and IGKV3-20*01. However, the frequencies of these IGKV usages were different between myeloblasts and mature myeloid cells (Tables 2 and 3). For example, IGKV3-20*01 was detected in 3/9 cases of monocytes and 2/3 cases of neutrophils (25/84 clones in total), and IGKV1-27*01 was detected in 2/9 cases of monocytes and 2/3 cases of neutrophils (24/84 clones in total). However, these two IGKV usages occurred with lower frequency in myeloblasts (IGKV3-20*01, 2/17 cases, 3/104 clones; IGKV1-27*01, 1/17 cases, 1/104 clones). On the other hand, IGKV1-5*03 was the most common IGKV used by CD33^+^ myeloblasts (14/17 cases, 64/104 clones), but it occurred only rarely in monocytes (2/9 cases, 11/84 clones) and was not detected in neutrophils. Of note is that IGKV1-NL1*01, which was observed frequently in AML cell lines (3/5 cases) and myeloblasts (3/17 cases), was absent in mature myeloid cells. These results suggest that IGKV3-20*01 and IGKV1-27*01 may be related to the function of mature myeloid cells, whereas IGKV1-5*03 and IGKV1-NL1*01 may have a role in leukemogenesis.

### Somatic hypermutation often occurs in the CDR regions of AML-derived IGKV

**Figure 4:**
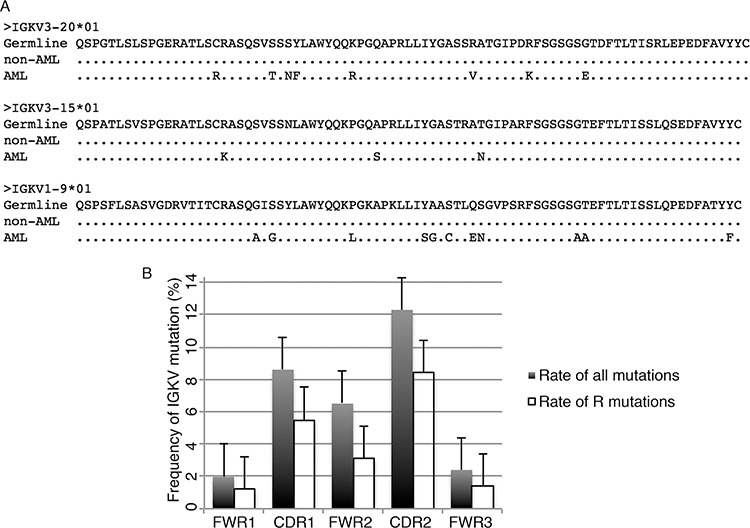
IGKV somatic hypermutation in myeloblasts and mature myeloid cells **A.** Alignment of three frequently used IGKV sequences (IGKV3-20*01, IGKV3-15*01 and IGKV1-9*01) from AML patients and patients with non-hematopoietic neoplasms (non-AML). The result showed that mutations frequently occurred in AML-derived IGKV but not in non-hematopoietic neoplasms-derived IGKV. **B.** The frequency of mutations in framework regions (FWR) and complementary determination regions (CDR). The mutation rate of all mutations and replacement mutation (R mutation) were presented.

It is known that somatic hypermutation frequently occurs in B-cell-derived IGKV upon antigen stimulation. We therefore studied the mutation status of myeloid cell-derived IGKV. Mutation status was designated as unmutated if there were < 2% mutations, or as mutated if there were ≥ 2% mutations compared with the germline sequences [18]. We found that somatic hypermutation occurred in all of the 14 clones of AML cell line-derived IGKV/IGKJ rearrangements assessed and 101 of 104 clones of primary myeloblasts. Moreover, within each set of IGKV/IGKJ rearrangement, such as IGKV1-5/IGKJ3 and IGKV1-NL1/IGKJ5, the mutation sites were identical even from different individuals (Supplementary Figure 1). Interestingly, somatic hypermutation was also detected in patients with non-hematopoietic neoplasms, but with a lower frequency [20/84 (24%) clones total; 17/61 clones of monocytes and 3/23 clones of neutrophils]. In addition, AML-derived IGKV demonstrated a higher mutation rate than that of non-AML-derived IGKV (median number of mutations, 8 versus 0) (Figure 4A).

Next, we studied the distribution of mutations in relation to certain hotspots, in particularly, the tetranucleotide motifs RGYW/WRCY (R = A/G, Y = C/T, W = A/T). Similar to B-cell-derived IGK, both the replacement (R) mutation sites and all mutation sites of AML-derived IGK were mainly located in the complementarity determination regions (CDR) rather than framework regions (FWR) (Figure 4B). However, the essential mutation targets of the RGYW (AGCT, AGTT, AGCA, AGTA) and WRCY (GGTT, GGTA, GGCT, GGCA) motifs, which have been identified as major mutation hotspots in B-cell-derived IGKV [19], was nearly absent in AML-derived IGK, with only 14/760 (1.8%) of all mutation sites occurred in the RGYW or WRCY motifs. Since somatic hypermutation is generally considered to be classical when >25% of the mutation sites occur in the RGYW or WRCY motifs [19], the AML-derived IGK therefore employed a different mechanism than classical somatic hypermutation.

### IGK expression does not affect AML cell proliferation, but is essential for cell migration

**Figure 5:**
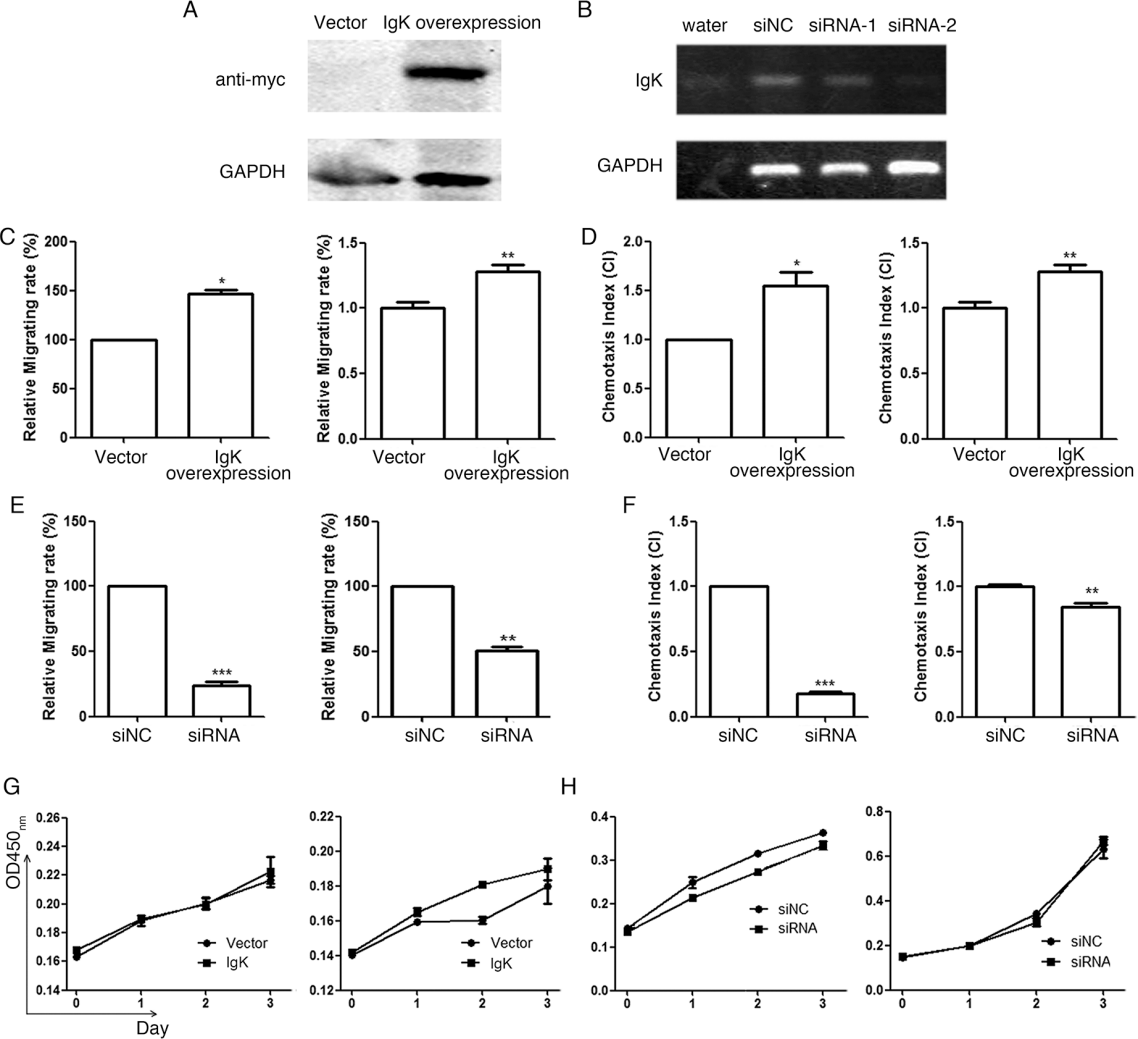
The effect of IGK expression on AML cell migration, chemotaxis and proliferation **A.** Western blot analysis with anti-myc antibody showed IGKV1-5*03/IGKJ3*01 (with myc tag) overexpression after transfecting the expression plasmid into HL-60 cell line. **B.** RT-PCR analysis showed that siRNA-2 had a much higher efficiency to inhibit IGK expression than siRNA-1. siRNA-2 was therefore used in the following knockdown experiments. Water and siNC were used as negative controls. **C.** The relative migrating rate of HL-60 (left) and NB4 (right) with IGK overexpression, using empty vector as the control. **D.** The chemotaxis index (CI) of HL-60 (induced by fMLP, left) and NB4 (induced by CXCL12, right) with IGK overexpression, using empty vector as the control. **E.** The relative migrating rate of HL-60 (left) and NB4 (right) with IGK knockdown by siRNA-2, using siNC as the control. **F.** The chemotaxis index (CI) of HL-60 (induced by fMLP, left) and NB4 (induced by CXCL12, right) with IGK knockdown using siRNA-2, using siNC as the control. **G.** The proliferation curves of HL-60 (left) and NB4 (right) with IGK overexpression, using empty vector as the control, assessed by CCK-8 assay. **H.** The proliferation curves of HL-60 (left) and NB4 (right) with IGK knockdown, using siNC as the control, assessed by CCK-8 assay.

To address if IGK expression is involved in cell proliferation and migration, we constructed an expression vector containing IGKV1-5*03/IGKJ3*01 sequence, the IGKV/IGKJ rearrangement which was most frequently found in myeloblasts in our study (Figure 5A). We also knocked down IGK expression with specific siRNA targeting the constant region of the IGK (Figure 5B). The vectors were then transfected into HL-60 and NB4 cell lines. Our results showed that IGKV1-5*03/IGKJ3*01 overexpression significantly promoted cell migration (Figure 5C), as well as fMLP, a chemopeptide, mediated chemotaxis of HL-60 cell line and CXCL12, a chemokine, mediated chemotaxis of NB4 cell lines (Figure 5D). As expected, two specific siRNAs for the constant region of the IGK inhibited the migration or chemotaxis motility (Figures 5E and 5F). However, IGKV1-5*03/IGKJ3*01 overexpression or knockdown did not affect cell proliferation of either HL-60 or NB4 cell lines (Figures 5G and 5H). These results suggest that IgK is mainly involved in the migration, but not proliferation, of leukemic cells.

## DISCUSSION

There have been a few reports of Ig gene expression in bone marrow or peripheral blood samples from AML patients [20]. However, the frequency of Ig expression in the previous reports was much lower compared to that detected in our current study. More importantly, it was not known if the Ig was produced by myeloid-derived cells, rather than being produced by residual B-cells that were present in the samples. It was also not clear if Ig was transcribed and translated by the myeloid cells or if it was due to non-specific binding of Ig produced by residual B-cells. Recently, we have reported that Ig γ and μ chains were both produced in AML cells and/or mature myeloid cells, which confirmed that Ig could indeed be produced by myeloid cells [16, 17]. Furthermore, the Ig γ chain was only found in AML cells, but the Ig μ chain was also found in mature monocytes and neutrophils from both patients with non-hematopoietic diseases and healthy donors, which demonstrated the difference in expression profile of different Ig subtypes.

Here we studied IGK expression in sorted CD33^+^CD19^−^CD138^−^ myeloblasts from 18 AML patients, and monocytes and neutrophils from 12 patients with non-hematopoietic neoplasms and 8 healthy individuals. After confirming that there were no residual B-cells or plasma cells in the samples by flow cytometry analysis, we detected the IGKV/IGKJ transcripts in CD33^+^ myeloblasts from all 6 AML cell lines and 17/18 AML patients, but not or only rarely in neutrophils (0/8) and monocytes (1/8) from healthy individuals. However, IGKV/IGKJ rearrangements were also detected in monocytes (11/12) and neutrophils (3/12) from patients with non-hematopoietic neoplasms. We do not know the significance of the latter finding, but one explanation may be that the IGKV/IGKJ rearrangements in mature monocytes and neutrophils from patients with non-hematopoietic neoplasms may be induced by certain tumor-related factors. Overall, these results show that IGK can be transcribed and expressed by myeloid cells under certain pathologic conditions.

The frequency of IGK expression detected in this study was much higher than that of IGG and IGM genes that have been report by us previously [16, 17]. We do not have a good explanation for this. However, it is well known that Ig light chains can be present in free forms under many pathologic conditions, such as amyloidosis, light chain deposition disease, inflammation, autoimmune diseases and monoclonal gammopathy of undetermined significance, and may play a role in the pathogenesis of these diseases [21, 22]. It is possible that myeloid derived-IgK may also be present as a free form. Another explanation is that it may be coupled with other subclasses of Ig heavy chain, such as α or δ chain.

In addition, we also assessed Ig lambda light chain (IGL) expression along with IGK in sorted myeloblasts in another group of AML patients (*n* = 12) by RT-PCR. Interestingly, we found that 5 patients expressed both IGK and IGL, 3 patients expressed IGL only, 1 patient expressed IGK only, and 3 patients did not express IGK or IGL (data not shown). This suggests that either IGK or IGL light chain, or both, can be expressed in myeloblasts of AML patients. Furthermore, we studied light chain expression in B-cells from a small group of leukemic patients (*n* = 12) by flow cytometry and found that the B-cells are polytypic for kappa and lambda expression.

Subsequently, we assessed sequence characters of myeloid-derived IGKV/IGKJ rearrangements, and found that, unlike that in B-cells from the same patients (which showed a polyclonal pattern), myeloid-derived IGKV/IGKJ rearrangements displayed unique monoclonal or oligoclonal IGK repertoire. Only 15 IGKV/IGKJ rearrangement patterns were observed in a total of 104 clones of myeloblasts assessed, and only 12 IGKV/IGKJ rearrangement patterns were found in 84 clones of mature myeloid cells from patients with non-hematopoietic neoplasms. Moreover, myeloblasts and mature myeloid cells showed differential preference in IGKV/IGKJ usages. Therefore, our results demonstrated an unique biased usage of IGKV repertoire in myeloid cells, which is in contrast to the IGKV repertoire seen in normal B-cells, B-lymphoma cells [23–26], and autoimmune diseases [27, 28] (Supplementary Figure 2). Interestingly, myeloblast-derived IGK displayed a high rate of somatic hypermutation, whereas only rare mutation was detected in monocytes or neutrophils-derived IGK. These results suggest that AML-derived IgK may be involved in leukemogenesis and/or AML progression.

To address the functional significance of IGK expression, we constructed an expression vector containing IGKV1-5*03/IGKJ3*01 which was most frequently found in myeloblasts in our study, and transfected it into HL-60 and NB4 cell lines. We found that, unlike AML-derived IgG or IgM, which could promote cell proliferation and survival [16, 17], expression of IGKV1-5/IGKJ3*01 did not affect the proliferation of AML cells. Instead, it significantly promoted migration and chemotaxis of the two AML cell lines assessed. We further confirmed the effect of IGK expression on cell migration and chemotaxis by knocking down IGK expression which resulted in a decrease of migration of these two AML cell lines.

In summary, we have shown that IGK gene is transcribed and expressed in AML cells, as well as monocytes and neutrophils from patients with non-hematopoietic neoplasms, but not or only rarely in myeloid cells from healthy individuals. Myeloid derived-IGK has unique IGKV/IGKJ sequences, and somatic hypermutation occurs preferentially in AML-derived IGK. More importantly, myeloid-derived IgK can promote migration and chemotaxis of AML cells. These findings suggest that myeloid-derived IgK may play a role in leukemogenesis and/or AML progression, and that it may serve as a tumor marker for monitoring minimal residual disease and developing target therapy.

## MATERIALS AND METHODS

### Cell lines and patient samples

AML cell lines, HEL, HL-60, KG-1, NB4, OCI-AML3 and THP-1, and B-cell line, SP53, were provided by MD Anderson Cancer Center. Peripheral blood specimens were collected from 18 AML patients, 12 patients with non-hematopoietic neoplasms and 8 healthy individuals. The study was conducted according to an institutional review board-approved protocol.

### Flow cytometry cell sorting and immunocytochemistry

Peripheral blood mononuclear cells were isolated by Ficoll-Hypaque density gradient centrifugation (for myeloblasts from AML patients) or after lysis of red blood cells (for monocytes and neutrophils from patients with non-hematopoietic neoplasms and healthy individuals), washed, stained with monoclonal antibodies CD19-APC, CD33-FITC and CD138-PE (BD Pharmingen, San Diego, USA), and sorted using a flow cytometer [16].

Indirect immunocytochemical staining was performed by incubating cytospin slides of AML cell lines with monoclonal anti-human IgK at 37°C for 45 minutes. After washing, the slides were incubated with anti-mouse IgG-horseradish peroxidase (Dako, Carpinteria, USA) at room temperature for 20 minutes, washed, and bound antibodies were detected using 3,3”-diaminobenzidine tetrahydrochloride (Dako) [16].

### Detection and sequencing analysis of IGKV/IGKJ transcripts

Total RNA was extracted from AML cell lines using Trizol reagent (Invitrogen, Carlsbad, USA) or sorted CD33^+^CD19^−^CD138^−^ cells using RNeasy Micro Kit (Qiagen, Chatsworth, USA), and reverse transcribed (RT) using Sensiscript RT Kit (Qiagen). IGKV/IGKJ transcripts were detected by a nested polymerase chain reaction (PCR) using upstream primers Vκ1 (5′-GACATCGAGCTCACCCAGTCTCC-3′) and Vκ2 (5′-GAAATTGAGCTCACGCAGTCTCCA-3′) and downstream primer Cκ1 (5′-TGGTGCAGCCACAGTT CGTTT-3′) in the first round, and same upstream primers and downstream primer Cκ2 (5′-CGGGAAGATGAAGACAGATGGTGC-3′) in the second round. The products were purified with DNA purification columns (Qiagen), ligated into pCR2.1 vector (Invitrogen), and transformed into DH5α competent bacteria (Invitrogen). 5–10 colonies per sample were chosen randomly and sequenced using T7 primer (Invitrogen).

### Cell proliferation, migration and chemotaxis assays

We constructed expression vectors with IgKV1-5*03/IGKJ3*01 sequences (to overexpress IgK) and synthesized siRNAs that target IgK C-region sequences (to knock down IgK expression), and transfected them into AML cell lines (Supplementary Methods). We assessed the effect of IgK overexpression or knockdown on cell proliferation using Cell Counting Kit-8 (Dojindo Molecular Technologies, Kumamoto, Japan) as described previously [16, 17].

For cell migration assay, we plated transfected HL-60 and NB4 cells at 5 × 10^5^ cells/well in 250μl RPMI 1640 without fetal bovine serum into the top chambers of a transwell plate with 8 μm pores (Corning, Corning, USA), and added RPMI 1640 with 10% fetal bovine serum into the lower chambers. The plates were incubated with 5% CO_2_ at 37°C for 48–72 h, and the cells in the lower chambers were counted by flow cytometry.

For chemotaxis analysis, transfected HL-60 and NB4 cells were plated as described above except that the lower chambers also contained 100 ng/ml fMLP (for HL-60) or 400 ng/ml CXCL12 (for NB4). After incubating for 0.5–2 h, the cells in the lower chambers were counted.

### Statistical analyses

Statistical analyses were performed using SAS 8.1 (SAS Institute Inc., Cary, USA). Mann-Whitney test was used to assess the differences between various groups. All tests were two-sided. A *p*-value of < 0.05 was considered statistically significant.

## SUPPLEMENTARY MATERIALS AND METHODS, FIGURES


