# Supplementary material for: IGK with conserved IGKV/IGKJ repertoire is expressed in acute myeloid leukemia and promotes leukemic cell migration
Source: Oncotarget. 2015 Sep 29;6(36):39062–72. doi: 10.18632/oncotarget.5393 (PMC4770757; doi:10.18632/oncotarget.5393)
Supplement: Supplementary file 1 [file oncotarget-06-39062-s001.pdf]

SUPPLEMENTARY MATERIALS AND METHODS

CONSTRUCTION OF EXPRESSION VECTOR AND TRANSFECTION

IGKV1-5*03/IGKJ3*01 full length sequence (with C region) with 10 mutations found in AML patients were cloned into the pcDNA3.1 myc-His (-) B vector (Invitrogen) using the primer sets BamHI- IGKV1-5*03 (5′-GGATCCATGGTGTTGCAGACCCAGGTCTT-3′) and IGKJ3*01-HindIII (5′-AAGCTTCCACACTCTCCCCTGTTGAAGCTC T-3′). For overexpression of IGK, the plasmid containing IGKV1-5*03/IGKJ3*01 sequence was transfected into HL-60 and NB4 cell lines by electroporation according to the manufacturer’s instructions (Nucleofector 2b Device, Lonza, Switzerland).

KNOCKDOWN OF IGK EXPRESSION BY SIRNA

siRNAs that target IGK C region sequence and non-specific siRNA (siNC, used as a negative control) were synthesized by Shanghai GenePharma Company (Shanghai, China). The sequences were as follows: siRNA-1, 5′-UCACAGAGCAGGACAGCAA-3′; siRNA-2, 5′-AGGCCAAAGUACAGUGGAA-3′; and siNC, 5′-GUAUGACAACAGCCUCAAGTT-3′. For knockdown of IGK expression, siRNA or siNC was transfected into HL-60 and NB4 cell lines by electroporation according to the manufacturer’s instructions (Nucleofector 2b Device).

A IGKV1-5*03/IGKJ3*01

```
             <---FWR1--><CDR1><------FWR2-----><C><---------------FWR3---------------><-CDR3><---Jκ3---->
Germline     GDRVTITCRASQSISSWLAWYQQKPGKAPKLLIYKASSLESGVPSRFSGSGSGTEFTLTISSLQPDDFATYYCQQYNSWIFTFGPGTKVDIK
Patient 1    ..K.I.S........D................M..V.T.Q....................................ST..............
Patient 2    ..K.I.S........D................M..V.T.Q....................................ST..............
Patient 3    ..K.I.S........D................M..V.T.Q....................................ST..............
Patient 4    ..K.I.S........D................M..V.T.Q....................................ST..............
Patient 5    ..K.I.S........D................M..V.T.Q....................................ST..............
Patient 6    ..K.I.S........D.....L..........M..V.T.Q....................................ST..............
Patient 7    ..K.I.S........D................M..V.T.Q....................................ST..............
Patient 8    ..R.I.SR.......D................M..V.T.Q....................................ST..............
```

B IGKV1-NL1*01/IGKJ5*01

```
             <---FWR1--><CDR1><------FWR2-----><C><---------------FWR3---------------><-CDR3><---Jκ5---->
Germline     GDRVTITCRASQGISNSLAWYQQKPGKAPKLLLYAASRLESGVPSRFSGSGSGTDYTLTISSLQPEDFATYYCQQYYSTPITFGQGTRLEIK
Patient 1    ............D..T.V....HR..........G.........N........A......................H...............
Patient 2    ............D..T.V....HR..........G.........N........A......................H...............
Patient 3    ............D..T.V....HR..........G.........N........A......................H...............
HEL          ............D..T.V....HR..........G.........N........A......................H...............
NB4          ............D..T.V....HR..........G.........N........A......................H...............
KG-1         ............D..T.V....HR..........G.........N........A......................H...............
```

Supplementary Figure S1: Alignment of IGK sequences with IGKV1-5*03/IGKJ3*01 and IGKV1-NL1*01/IGKJ5*01 rearrangements from AML patients and AML cell lines and the germline sequence.

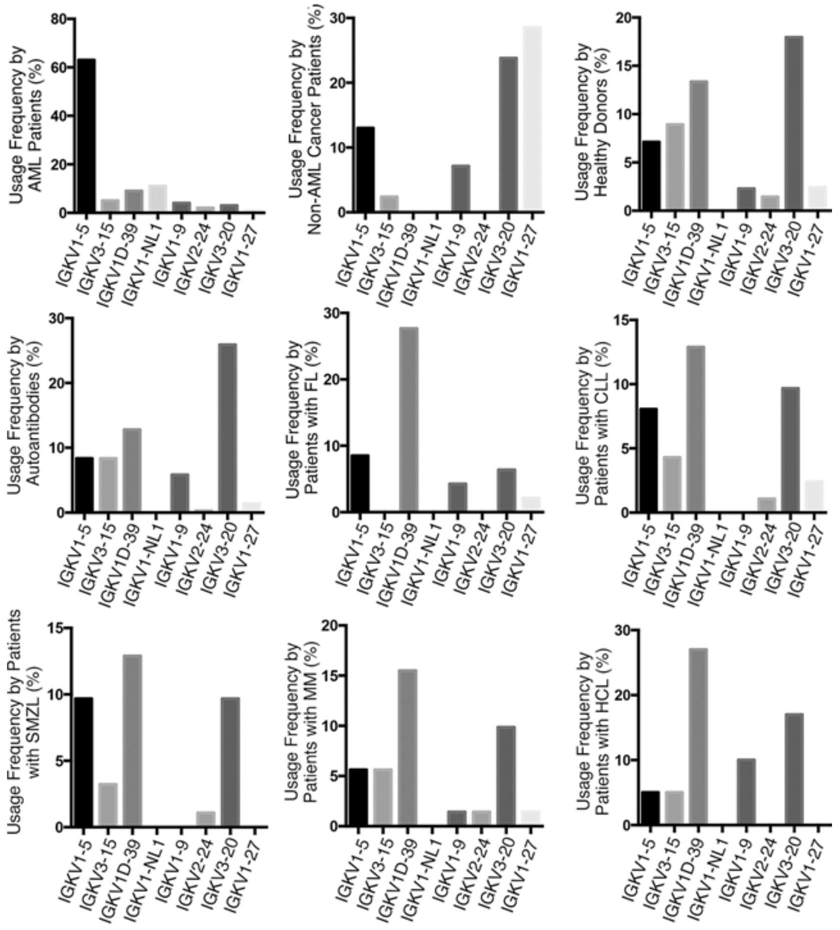

Supplementary Figure S2: Comparison of IGKV usage frequency among patients with acute myeloid leukemia (AML), non-hematopoietic neoplasms (non-AML), autoimmune disease, follicular lymphoma (FL), chronic lymphocytic leukemia (CLL), splenic marginal zone lymphoma (SMZL), multiple myeloma (MM), hairy cell leukemia (HCL), and healthy donors.
